# *Armigeres subalbatus* is a potential vector for Zika virus but not dengue virus

**DOI:** 10.1186/s40249-022-00990-0

**Published:** 2022-06-04

**Authors:** Wenqiang Yang, Siyu Zhao, Yugu Xie, Tong Liu, Ling Kong, Yijia Guo, Zhensheng Xie, Peiwen Liu, Xiao-Guang Chen

**Affiliations:** Department of Pathogen Biology, Institute of Tropical Medicine, School of Public Health, Southern Medical University, Guangzhou, China

**Keywords:** *Armigeres subalbatus*, Zika virus, Dengue virus, Vector

## Abstract

**Background:**

Zika virus (ZIKV) and dengue virus (DENV) are closely related flaviviruses primarily transmitted by *Aedes* mosquitoes. *Armigeres subalbatus* is an emerging and widely distributed mosquito, and ZIKV has been detected and isolated from it. However, it is not clear whether *Ar.* s*ubalbatus* could be a vector for ZIKV and DENV or not. In this study, we investigated the infection and transmission of *Ar. subalbatus* to ZIKV and DENV.

**Methods:**

A line of *Ar. subalbatus* was isolated from Guangdong, China, and further identified by the mitochondrial cytochrome oxidase subunit 1 (*COI*) gene. The adults of *Ar. subalbatus* were fed with blood meal containing ZIKV or DENV-2. At 4, 7, 10, 14, and 21 days post-inoculation (dpi), the infections of ZIKV or DENV-2 in the midguts, ovaries and salivary glands were detected and quantified by RT-PCR and RT-qPCR. To assess the transmissibility, suckling mice were exposed to bites of ZIKV-infected mosquitoes, and ZIKV was detected in brain tissue by RT-qPCR and plaque assays. Furthermore, the larvae of *Ar. subalbatus* were reared in artificial urine containing ZIKV or DENV-2. The infection rates and viral titers of larvae and adults were analyzed by RT-PCR and RT-qPCR, and the viral distribution in larval tissues was observed by immunohistochemistry. Chi-square test and one-way ANOVA analysis were used for assessing the infection rate and viral titer in varied tissues and different time points, respectively.

**Results:**

Following oral inoculation, ZIKV but not DENV-2 could be detected in *Ar. subalbatus* midguts at 4 dpi, ovaries at 7 dpi and salivary glands at 10 dpi. The highest infection rate (IR) of ZIKV was 27.8% in midgut at 7 dpi, 9.7% in ovary and 5.6% in salivary gland at 21 dpi. Eight days after being bitten by ZIKV-positive mosquitoes, ZIKV was detected in three brain tissues out of four suckling mice exposed to bites. ZIKV could be detected in the larvae reared in artificial urine contained ZIKV at a high concentration of 10^5^ pfu/ml and various tissues of adults with a low infection rate (0.70–1.35%). ZIKV could be observed in anal papillae and midgut of larvae at 4 dpi under laboratory conditions.

**Conclusions:**

ZIKV but not DENV-2 can infect *Ar. subalbatus* by blood meal and artificial urine, and the infected mosquitoes can transmit ZIKV to suckling mice by bite. From these findings, we can conclude that the *Ar. subalbatus* isolated from Guangdong province, China, is a potential vector for ZIKV and should therefore be considered in vector control programs to prevent and control of Zika virus disease.

**Graphical Abstract:**

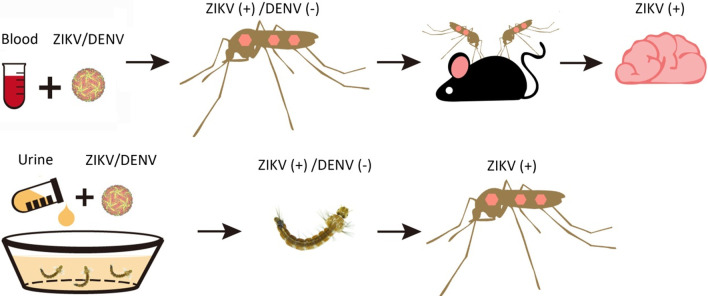

**Supplementary Information:**

The online version contains supplementary material available at 10.1186/s40249-022-00990-0.

## Background

Zika virus (ZIKV) is a mosquito-borne flavivirus which causes Zika virus disease (Zika) with a range of symptoms, including serious neurological complications [1–3]. ZIKV was first isolated from a febrile sentinel rhesus monkey in 1947 in Uganda, Africa, and from an *Aedes africanus* within the same forest in 1948 [4, 5], which indicated that there existed the sylvatic cycle of ZIKV. The first large Zika outbreak was on the Micronesian island of Yap in 2007 [6], and an extensive spread in the French Polynesia followed in 2013–2014 [7]. Severe Zika outbroke in Brazil in 2015 with increase cases of microcephaly, and the epidemic rapidly spread to more than 30 other countries as of February 29, 2016 [8, 9]. WHO classified the epidemic as a “public health emergency of international concern” (PHEIC) [10]. The first case of Zika was imported in the mainland of China in 2016 [11]. Since then, at least 22 cases have been reported in Guangdong, Zhejiang and other provinces of China [12, 13]. As of July 2019, 87 countries and territories had the evidence of autochthonous mosquito-borne transmission of ZIKV [14]. Dengue virus is another flavivirus and causes dengue fever which is a rapidly emerging mosquito-borne infectious disease and now endemic in over 128 countries and regions around the world [15]. South China especially Guangdong has often experienced dengue epidemics and had an outbreak in 2014 which documented more than 45,000 infections [16]. *Aedes aegypti* and *Ae. albopictus* are currently considered to be the major vectors for ZIKV and DENV [17–19].

*Armigeres subalbatus* (Diptera: Culicidae) adults can inhabit houses, livestock sheds and wild grass fields [20]. Larvae mainly develop in bamboo tubes, tree holes, dilute cesspools, sewage pits, sewers, and standing water in containers [20]. *Armigeres subalbatus* has been reported from many countries around the world, such as Japan, Thailand, and Malaysia [20–23]. In China, the mosquito is mainly distributed south of the Yangtze River [20]. *Armigeres subalbatus* has been proved to be a vector for a variety of pathogens including parasites and viruses, such as Filaria and Japanese encephalitis virus [24, 25]. Recent investigations showed that ZIKV could be isolated from the wild-caught *Ar. subalbatus* in Guizhou Province of China [26] or detected from the wild-caught *Ar. subalbatus* in Thailand [27]. A laboratory strain of *Ar. subalbatus* had been proved to be susceptible to ZIKV and the infected mosquitoes could transmit virus to suckling mice by bite [28]. Moreover, Du et al. revealed that *Ae. aegypti* could be infected by ZIKV either by blood meal or from the aquatic environments with infectious urine [29]. In addition, *Ar. subalbatus* in Taiwan province had been documented as one of the vectors of dengue fever [20, 30], however, such reports cannot be checked in any peer-reviewed journals. At present, it is not clear whether *Ar. subalbatus* could be a vector for ZIKV and DENV or not.

In this study, we explored the susceptibility of *Ar. subalbatus* isolated in Guangdong Province, China to ZIKV and DENV and its ability to transmit these viruses in the laboratory setting. Furthermore, we investigated the possibility of the larvae infection by ZIKV and DENV-2 from the artificial viral urine, and the route by which the virus invades larvae.

## Methods

### The establishment of *Ar. subalbatus* laboratory strains

*Armigeres subalbatus* were collected from a campus (23°12′03.1′′N, 113°17′20.1′′E) located in Guangzhou City, Guangdong Province, China. Representative morphology and *COI* gene sequencing confirmed the identity of *Ar. subalbatus* (Additional file 1: Fig. S1, Additional file 2: Table S1) [20, 31]. All mosquitoes were maintained under standard insectary conditions of 27 ± 1 °C, 70–80% relative humidity, and a light: dark cycle of 16 h:8 h [17]. The larvae of *Ar. subalbatus* were reared in stainless steel trays (24 cm × 34 cm × 6 cm) and fed daily with turtle food (INCH-GOLD^®^). After pupation, the pupae were picked with a pipette into a 300-ml plastic cup containing 200 ml of dechlorinated water and then transferred into mosquito cages (25 cm × 25 cm × 35 cm), and adults of *Ar. subalbatus* were fed on 10% glucose water.

### Cell line and virus

C6/36 cells were cultured in RPMI-1640 medium supplemented with 10% heat inactivated fetal bovine serum (FBS) and maintained at 28 ℃.

ZIKV (GenBank Acceptance No. KU820899.2) was provided by the Centers for Disease Control of Guangdong Province, China, which was isolated from a ZIKV patient in Zhejiang Province of China in 2016 and classified as the Asian lineage [32, 33]. The virus had been passaged once via intracranial inoculation of suckling C57 mice and twice in C6/36 cells. ZIKV were collected after 5–7 days of enrichment in C6/36 cells at 28 ℃ and stored at − 80 ℃.

DENV-2 (New Guinea C, GenBank: AF038403.1) was provided by the Key Laboratory of Tropical Disease Control of Sun Yat-sen University (Guangzhou, China), and had been passaged one generation in our lab. DENV-2 were collected after enrichment in C6/36 cells at 37 ℃ for 36–48 h until obvious cytopathic effects were observed and stored at − 80 ℃.

ZIKV and DENV-2 supernatant were harvested and the titers were ready to be quantified with plaque assay before being blood-fed to the mosquitoes.

### Oral infection of mosquitoes

The ZIKV and DENV-2 supernatant with the viral titer of 10^6^ pfu/ml were mixed with defibrinated sheep blood at a ratio of 2:1. *Armigeres subalbatus* had been passaged to the 12th generations in laboratory. Female adults aged 3–5 days post-emergence were anesthetized with CO_2_ and transferred into cages (60–70 females per pool). Female mosquitoes were starved for 20–24 h and allowed to feed on the blood meal maintained at 37 ℃ for 1 h with a Hemotek blood feeding system (Discovery Workshops, Lancashire, UK). Fully engorged females were selected and incubated at 28 ℃, 80% relative humidity, and a light: dark cycle of 16 h:8 h and fed on 10% glucose water for viral detection. The midgut, ovaries, and salivary glands were dissected to detect ZIKV at 4, 7, 10, 14 and 21 dpi or DENV-2 at 4, 7, 10 and 14 (24 females at each time point).

### Horizontal transmission assays

After 10 days of the ZIKV-feeding blood meal, parts of the leg of females were used to detect ZIKV by RT-PCR. The ZIKV-positive females were selected and divided into four groups (10–11 females per group). These females continued to be reared for two days and then starved for 18–20 h. Each group was allowed to bite one 4-day-old suckling mice for 2 h. All the suckling mice were euthanized at 8 day post mosquitoes feeding and the brain of each individual mouse was dissected to detect the virus titer by RT-qPCR and plaque assay.

### Urine infection

1 ml of ZIKV supernatant was mixed into 200 ml clean water containing 0.5 ml urine as artificial viral urine (single addition, final titers was 10^5^ pfu/ml). The viral activity of ZIKV in artificial viral urine was detected by RT-qPCR at 0 h, 24 h and 48 h. Every 24 h until pupation, 1 ml of ZIKV and DENV-2 (final titers was 10^4^ pfu/ml) supernatant was added into 200 ml clean water containing 0.5 ml urine, respectively as artificial virus urine (continuous addition).

The larvae of *Ar. subalbatus* (200 larvae per pool), in 33rd generation in laboratory were reared in the artificial viral urine at first, second and third instar, respectively. The larvae were reared to the fourth instar and then washed in clean water for 3 times (5 min per time) before ZIKV and DENV-2 detection. 8–12 days after the emergence of larvae reared in artificial virus urine (continuous addition, final titers was 10^5^ pfu/ml), the midgut, ovaries and salivary glands of 426 females were dissected to detect ZIKV in adult stage.

### The route of ZIKV infection

1 ml of ZIKV supernatant was mixed into 20 ml clean water containing 50 μl urine as artificial viral urine (single addition, final titers was 10^6^ pfu/ml). Third instar larvae (160 larvae per pool) were reared in the artificial viral urine. 16 pools of the urine-infected larvae (10 larvae per pool) were collected at 1, 2, 3 and 4 dpi for ZIKV detection. Meanwhile, the midgut, anal papillae and carcass of 37 pools of larvae (10 larvae per pool) were dissected at 4 dpi for the route of ZIKV infection. The remaining of larvae were washed with clean water and allowed to remove part of anal papillae at 4 dpi to detected ZIKV by RT-PCR. ZIKV-positive larvae were fixed in 4% formaldehyde solution for Immunohistochemistry assay.

### Saliva collection

ZIKV-positive female mosquitoes after 14 days of oral infection were anesthetized with CO_2_. The legs and wings of adults were removed with dissecting scissors, and the mosquito's proboscis was then inserted into a pipette tip containing 20 μl of FBS for 30 min to collect mosquito saliva. Afterward, the saliva was placed into a 1.5-ml RNase-free EP tubes containing 180 μl of Dulbecco's modified eagle medium (DMEM) and stored at −80 ℃.

### ZIKV and DENV-2 detection and quantification

Total RNA of collected samples were extracted according to the manufacturer’s protocol with TRIzol reagent and dissolved in 20 µl of RNase-free water. cDNA synthesis was performed by using the GoScript Reverse Transcription System (Promega, Madison, WI, USA), reverse specific primer (ZIKV) [17] or random primer (DENV-2).

Reverse transcriptase-PCR (RT-PCR) was used to detect the viral genome in tissues of adults and larvae, following previous protocol [17]. Absolute quantitative real-time PCR (RT-qPCR) was used to quantify the viral copies of the positive sample following previous protocol [17, 18]. A standard curve for ZIKV detection was established by tenfold dilutions of the plasmid standard (5.73 × 10^3^–5.73 × 10^7^ copies/μl). A standard curve for DENV-2 detection was established by tenfold dilutions of the plasmid standard (1.83 × 10^3^–1.83 × 10^7^ copies/μl). RNA copies from each sample were quantified by comparing the cycle threshold value with the standard curve. These experiments were conducted according to standard procedures in a biosafety level 2 laboratory.

### Plaque assay

BHK-21 cells were suspended in 10% FBS in DMEM and plated in 12-well plates. The cells were incubated in a cell culture incubator until 90% to 95% confluency was reached. The saliva of female mosquitoes and supernatants of the mouse brain were inoculated into the well individually with serial dilutions. After incubating cells and viruses in a cell incubator for 1 h, the cell culture medium was removed from the wells, and the cells were washed twice with phosphate-buffered saline (PBS). Monolayers of cells inoculated with the virus were covered with methylcellulose. Six days after incubation, the cells were fixed in a 4% paraformaldehyde solution for 1 h and then stained with a crystal violet solution for 30 min at room temperature. The wells were washed with tap water and dried. The number of plaques in the wells was easily observed and counted. The plaque forming units (pfu/ml) per ml were calculated as follows: pfu/ml = average number of plaques/[(dilution factor for wells) (volume of inoculum per plate)].

### Immunohistochemistry assay

The entire body of ZIKV-positive larvae fixed in 4% formaldehyde solution was embedded in paraffin and serially cut for histologic examination. The slides were stained with rabbit monoclonal ZIKV NS1 protein antibody (Thermo Fisher) and anti-rabbit IgG secondary antibody (Abcam) [34], and the specimens were examined under a light microscope (Olympus, Japan). Larvae reared in artificial urine without ZIKV were used as the control.

### Statistical analysis

All statistical analyses were performed with SPSS 20.0 (IBM, Chicago, IL, USA). Chi-square test was applied to compare the infection rate (IR) of the same tissue at different time points. The *P* value significance was corrected by Bonferroni adjustments. The RNA copy number of ZIKV was first logarithmically converted, and then One-way ANOVA was used to compare the amount of ZIKV in the same tissue at different time point. *P* < 0.05 was considered statistically significant.

## Results

### *Armigeres subalbatus* can be infected with ZIKV but not DENV-2 by blood feeding

**Fig. 1:**
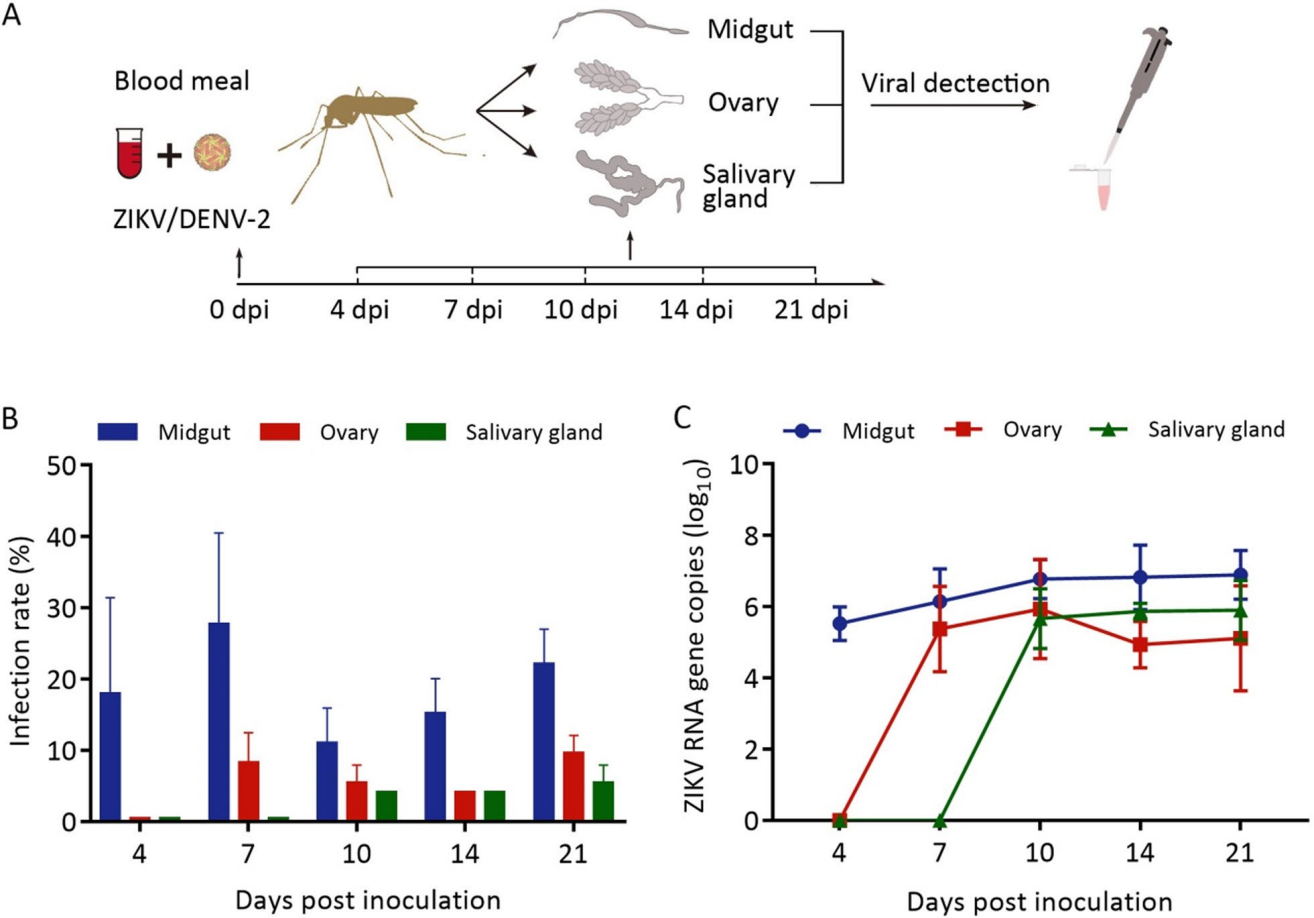
The blood-fed infection of ZIKV or DENV-2 in *Ar. subalbatus*. **A** Schematic diagram of oral infection experiment. **B** Infection rates and (**C**) RNA copies of ZIKV in various tissues at different days post inoculation (dpi). The results are present as the means ± *SD*. Error bars indicate *SD*s. The experiment was repeated three times. *SD*: Standard deviation.

Viral genome of ZIKV or DENV-2 of various tissues in adults was detected at different dpi (Fig. 1A). RT-PCR results showed that ZIKV could be initially detected in the midgut (18.0%) at 4 dpi, in the ovaries (8.3%) at 7 dpi and in the salivary glands (4.2%) at 10 dpi (Fig. 1B; Additional file 3: Table S2). We separately compared the midgut infection rate of 4, 7, 10, 14, and 21 dpi in *Ar. subalbatus*. The midgut infection rate at 4, 10, 14, and 21 dpi showed no significant differences (*χ*^2^ = 4.237, *P* > 0.05), but at 7 dpi is higher than the others four time points (*χ*^2^ = 10.313, *P* < 0.05) (Fig. 1B).

The highest titration (log_10_) of ZIKV was 6.90 ± 0.69 at 21 dpi in the midgut, 6.20 ± 1.34 at 10 dpi in the ovaries and 5.90 ± 0.84 at 14 dpi in the salivary glands (Fig. 1C). Compared with 4 dpi, the RNA copy number (log_10_) of ZIKV in midguts was significantly increase at 7, 10, 14 and 21 dpi (*t* = 13.214, *P* < 0.05) (Fig. 1C).

The amounts (log_10_) of ZIKV in ovaries (*t* = 0.507, *P* > 0.05) and salivary glands (*t* = 0.072, *P* > 0.05) showed no significant difference at different time point (Fig. 1C).

Additionally, thirteen saliva samples were collected from the salivary glands of ZIKV-positive mosquitoes, and the result showed that 7 saliva samples contained ZIKV, and the mean viral titer was 3.12 ± 0.64 log_10_ pfu/ml. These results showed that ZIKV could infect and overcome the midgut and salivary gland barriers of *Ar. subalbatus*. Positive salivary glands and saliva indicated that *Ar. subalbatus* has the potential risk of transmitting ZIKV to host organisms.

RT-PCR and RT-qPCR results indicated that DENV-2 was not detected in midguts, ovaries, and salivary glands of *Ar. subalbatus* females at 4 dpi, 7 dpi, 10 dpi and 14 dpi (24 adults per time point) (Additional file 4: Fig. S2). The result showed that the mosquito isolated from Guangdong province is not able to infect DENV-2.

### Infected *Ar. subalbatus* can transmit ZIKV to suckling mouse by bite

The results showed that three of the four suckling mouse brains were ZIKV-positive, and the virus titers (log_10_) ranged from 6.36 to 7.30 copies/ml by RT-qPCR or 2.4 to 4.0 pfu/ml by plague assay (Table 1). These data demonstrated that the infected *Ar. subalbatus* can transmit ZIKV to suckling mice by bite.

**Table 1:** Zika virus titer in the brain tissue of suckling mice

Group	No. of mosquitos with positive legs	No. of suckling mice	Zika virus titer in brain tissue	
			RT-qPCR (Log_10_, copies/ml)	Plaque assay (Log_10_, pfu/ml)
1	10	1	6.36	2.4
2	10	1	6.65	2.4
3	10	1	–	–
4	11	1	7.30	4.0

–: no ZIKV was detected

### Larvae of *Ar. subalbatus* can be infected by ZIKV but not DENV-2 in an artificial urine environment

**Fig. 2:**
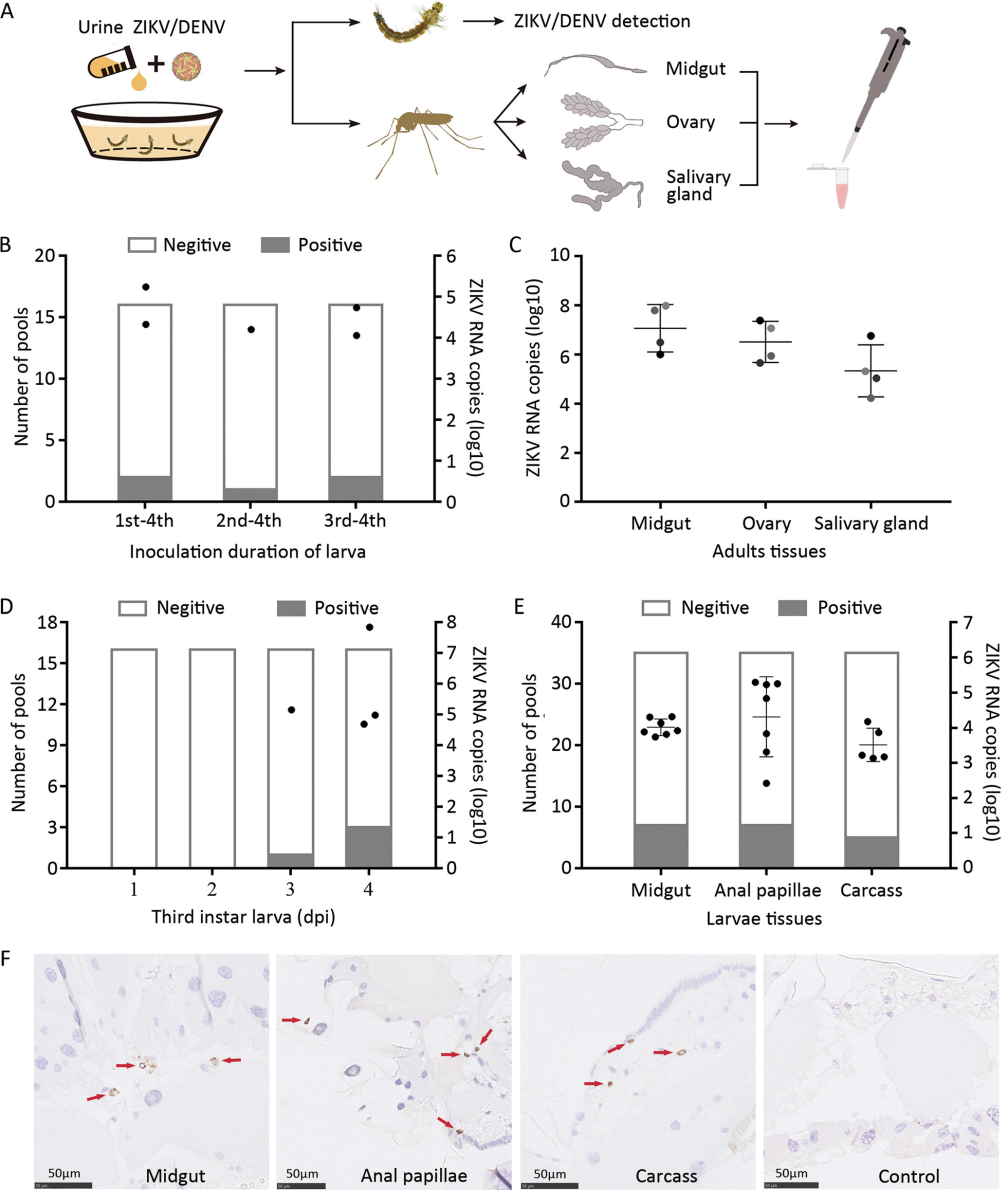
The artificial urine infection of ZIKV or DENV-2 in the larvae of *Ar. subalbatus*. **A** Schematic diagram of the artificial virus urine infection experiment. **B** Susceptibility of different instar larvae reared in artificial urine contained ZIKV, and virus was detected in the fourth instar larvae. **C** RNA copies of ZIKV in infected midguts, ovaries, and salivary glands of adults. The results are expressed as the means ± standard errors (SEs). **D** The third instar larvae were reared in artificial urine with ZIKV, and the infection of larvae was detected at 1, 2, 3, 4 days post inoculation (dpi) with RT-PCR and RT-qPCR. **E** ZIKV was detected in midgut, anal papillae and carcass of fourth instar larvae at 4dpi with RT-PCR and RT-qPCR. **F** Virus particles were detected with ZIKV NS1 protein antibody (Thermo Fisher) by IHC and are displayed as obvious brownish-red marked by red arrow

Viral genome in larvae and adults reared in artificial virus urine was determined (Fig. 2A). RT-PCR results showed ZIKV could not be detected in fourth instar larvae (39 pools) reared in artificial urine containing ZIKV in the single addition experiment (Table 2). ZIKV copies in artificial urine decreased to 4.7% at 24 h and 1.2% at 48 h compared to 0 h (Additional file 5: Fig. S3A), and the virus was added daily in artificial urine. In the continuous addition experiment, ZIKV could be detected in fourth instar larvae (38 pools) with a low infection proportion (4/38) (Table 2). The similar infection rates were observed in different instar stage (Fig. 2B). The viral RNA was also detected in various tissues of adults after eclosion for 8–12 days (Table 2; Fig. 2C). The copy number (log_10_) in salivary gland of adults ranged from 4.23 to 6.76 copies/µl (Fig. 2C).

**Table 2:** Infection rate of larvae for ZIKV in artificial urine

Infection method	Viral titer (pfu/ml)	Experiment repetition	No. of larvae in test pools^a^	No. of positive pools of larvae	Infection rate of adult mosquitoes^b^
Single addition	10^5^	1	13	0	–
		2	13	0	–
		3	13	0	–
Continuous addition	10^5^	1	16	2	1.35 (2/148)
		2	11	1	0.70 (1/143)
		3	11	1	0.74 (1/135)

^a^Ten larvae per pool

^b^Infection rate: No. positive mosquitoes/No. of tested mosquitoes (%)

The detection result of fourth instar larvae (41 pools) showed that the larvae of *Ar. subalbatus* could not be infected by DENV-2 in the continuous addition experiment. (Additional file 6: Table S3). These results indicate that larvae of *Ar. subalbatus* can be infected by ZIKV but not DENV in an artificial urine infection environment.

### ZIKV can infect larvae via the anal papilla and midgut

Total of 85 pools fourth larvae were dissected for in the continuous addition experiment, RT-PCR and RT-qPCR indicated that the midgut and anal papilla were positive in 7 of 85 pools, and carcass were positive in five pools (Additional file 5: Fig. S3B). The similar infection rate was observed in the midgut and anal papilla. We suspected that the method of continuous addition provided plenty of time for ZIKV to accumulate and spread in the larvae. Thus, it is difficult for us to analyze the route of the virus infects larvae.

Next, we intended to provide the larvae with a short time window for ZIKV inoculation by single addition, and detected ZIKV in early infected larvae. Given the larvae could not be infected by ZIKV in artificial virus urine (200 ml), we reared the larvae in a smaller artificial urine water (20 ml) containing ZIKV at a higher-dose. Due to similar infection rate in different instar (Fig. 2B) and larger size of larvae that was convenient for us to dissect various tissues, the third instar larvae were selected and reared in artificial urine containing ZIKV (single addition). RT-PCR and RT-qPCR showed that one ZIKV positive pool was detected at 3 dpi and three pools at 4 dpi (Fig. 2D). Considering the relatively high rate of infection and the experimental possibilities, the midgut, anal papilla, and carcass of larvae at 4 dpi were dissected for viral detection. RT-PCR and RT-qPCR also showed similar infection rates (7/35) in midgut and anal papilla (Fig. 2E).

The immunohistochemistry assay (IHC) was performed to further verify infection and dissemination of ZIKV in larvae. Viral particles were observed in midgut, anal papilla and other tissues (Fig. 2F). These findings revealed that ZIKV could infect the larvae of *Ar. subalbatus* via midgut and anal papilla.

## Discussion

According to findings above, we confirmed that *Ar. subalbatus* isolated from Guangdong province can be infected with and transmit ZIKV, but this was not observed for DENV. The larvae of *Ar. subalbatus* can acquire ZIKV via multiple routes, including midgut and anal papillae, and the ensuing adults have the potential to transmit ZIKV. One thing we should indicate that *Ar. subalbatus* used in this study had been passaged 12th generations in laboratory, which might generate some differences with the field population.

The international pandemic of Zika virus disease suggests that its vectors might be diverse, and there may be other vectors besides *Aedes* mosquitoes. *Armigeres subalbatus* is widely distributed, and previous studies have shown that the mosquito has the potential of infecting and spreading ZIKV [26–28]. Findings in this study are consistent with previous reports [28] and further suggest that *Ar. subalbatus* could be a neglected vector for Zika, that needs to be paid attention to in the prevention and control of Zika. Compared with *Ae. aegypti* and *Ae. albopictus*, the main vectors of ZIKV [18], there is a relatively low susceptibility to ZIKV in *Ar. subalbatus*.

Researches on Zika virus patients show that they can excrete high concentration of ZIKV through urine [35–37], estimated maximum viral load can reach to 2.2 × 10^8^ copies/ml [35], which may contaminate the water in the external environment, including the breeding places of mosquitoes. The larvae of *Ar. subalbatus* have the habit of breeding in such an environment. Du’s research shows that larvae of *Ae. aegypti* can be infected by ZIKV in polluted water [29]. Consider the larvae of vector mosquito could acquire ZIKV from the breeding place contaminated by patient’s urine once or many times, the methods of single and continuous addition were used to infect the larvae. This study confirmed that larvae of *Ar. subalbatus* can be infected by the virus from the artificial virus urine, and the infection can persist to the adult stage. The finding that *Ar. subalbatus* can be infected by ZIKV through breeding waters suggests that this could be a new mosquito-borne transmission route of Zika virus disease. In our study, a higher dose of DENV than reported in Andries’s study (10–10^5.2^ RNA copies/ml) was used [38]. Due to lower concentration of DENV in urine of patients, it is almost impossible for the larvae to be infected by the virus in the wild.

The midgut barrier is crucial for adult mosquitoes to prevent virus infection and spread. In artificial urine infection experiment, if oral infection is the only way for larvae to acquire the virus, we suspect that the midgut is preferentially infected by ZIKV and the infection rate in the midgut is higher than other tissues. However, beyond our expectations, the results of anatomical and immunohistochemistry assay showed that ZIKV could be detected in the midgut, anal papillae and other tissues in early infection with a similar infection rate, which implied that ZIKV invade the larvae via multiple routes. According to previous findings in our study, the primary infection site of mosquito densovirus in larvae of *Ae. albopictus* was the anal papillae, followed by bristle cells [39]. The anal papillae of mosquito larvae are osmoregulatory organs that are in direct contact with the external aquatic environment [40]. The present study indicated that anal papillae of *Ar. subalbatus* larvae are also responsible for the infection by ZIKV, which could be another route of infection besides the midgut.

Although *Aedes* mosquitoes are considered the primary vectors of ZIKV, the significance of *Ar. subalbatus* cannot be neglected considering its widespread distribution and preference for breeding in contaminated aquatic environments. However, the mosquito population and the virus isolate used in the present experiments may limit the results and findings from this study.

## Conclusions

A strain of *Ar. subalbatus* isolated from Guangdong, China, can infect and transmit ZIKV but not DENV-2 under laboratory conditions. Moreover, the larvae of *Ar. subalbatus* can also be infected by ZIKV contaminated urine. Because of the absence of vaccines and specific treatment, exploring the vector competence of *Ar. subalbatus* for ZIKV and DENV provides an experimental basis for vector control and the prevention and control of mosquito-borne viral diseases.

## Supplementary Information

**Additional file 1: Fig. S1**. The morphology of *Ar. subalbatus* isolated from Guangdong Province, China. (A) Female adult. (B) Male adult.

**Additional file 2: Table S1**. Genetic analysis of the mosquito species isolated in Guangdong based on the *CO*I gene.

**Additional file 3: Table S2**. The infections of ZIKV in *Ar. subalbatus*.

**Additional file 4: Fig. S2**. The blood-fed infection of DENV-2 in *Ar. subalbatus.* Infection rate (A) and RNA copies (B) of DENV-2 in infected midguts, ovaries, and salivary glands. The results are expressed as means ± standard errors (SEs). The experiment was repeated three times.

**Additional file 5: Fig. S3**. Infection rate of ZIKV in various tissues of fourth instar larvae (continuous addition). (A) ZIKV copies in artificial urine at different times. (B) ZIKV was detected in midgut, anal papillae and carcass of 4th instar larvae with RT-PCR and RT-qPCR.

**Additional file 6: Table S3**. Infection rate of DENV-2 in larvae of *Ar. subalbatus*.

## Data Availability

The data used in this study are available, if necessary; please contact the first author (WQY) or corresponding author (XGC).

## Abbreviations

ZIKV: Zika virus
DENV: Dengue virus
*CO*I: Cytochrome oxidase I
PBS: Phosphate-buffered saline
IHC: Immunohistochemistry
FBS: Fetal bovine serum
RT-PCR: Reverse transcription polymerase chain reaction
RT-qPCR: Quantitative reverse transcription polymerase chain reaction
RNA: Ribonucleic acid
cDNA: Complementary deoxyribonucleic acid
PFU: Plaque-forming unit
NS1: Nonstructural protein 1
RPMI: Roswell Park Memorial Institute
DMEM: Dulbecco’s Modified Eagle Medium
