# Supplementary figures and images for: Armigeres subalbatus is a potential vector for Zika virus but not dengue virus
Source: Infect Dis Poverty. 2022 Jun 4;11:62. doi: 10.1186/s40249-022-00990-0 (PMC9166152; doi:10.1186/s40249-022-00990-0)

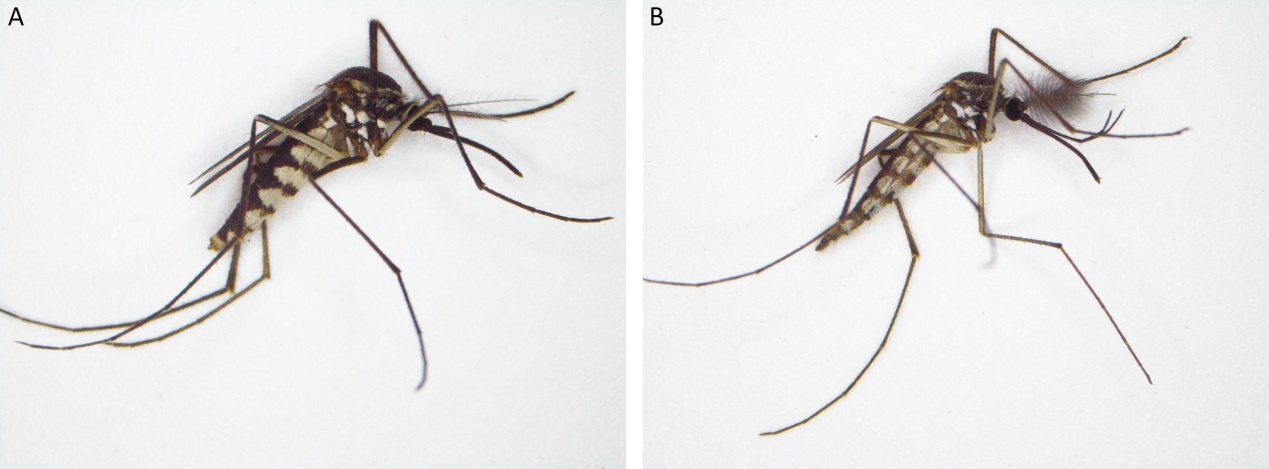

Supplement: Supplementary file 1 — Additional file 1: Fig. S1. The morphology of Ar. subalbatus isolated from Guangdong Province, China. (A) Female adult. (B) Male adult. [file 40249_2022_990_MOESM1_ESM.docx]

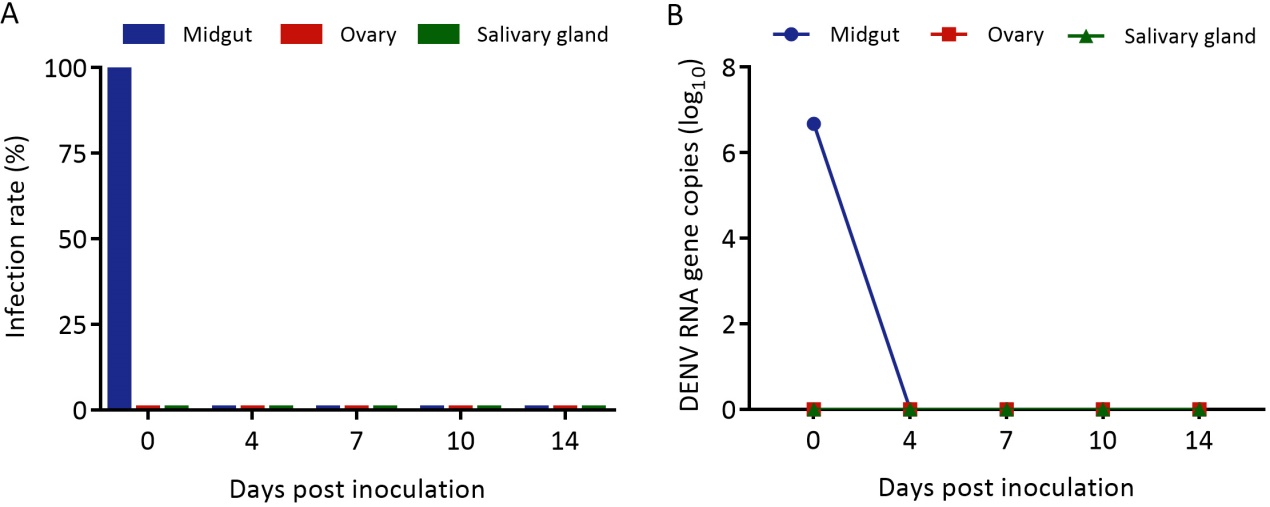

Supplement: Supplementary file 4 — Additional file 4: Fig. S2. The blood-fed infection of DENV-2 in Ar. subalbatus. Infection rate (A) and RNA copies (B) of DENV-2 in infected midguts, ovaries, and salivary glands. The results are expressed as means ± standard errors (SEs). The experiment was repeated three times. [file 40249_2022_990_MOESM4_ESM.docx]

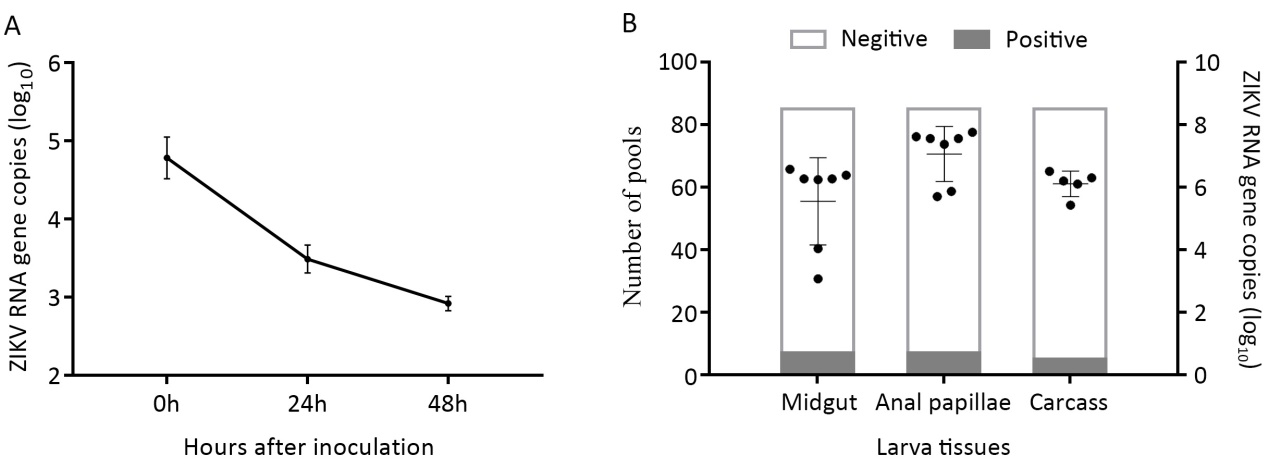

Supplement: Supplementary file 5 — Additional file 5: Fig. S3. Infection rate of ZIKV in various tissues of fourth instar larvae (continuous addition). (A) ZIKV copies in artificial urine at different times. (B) ZIKV was detected in midgut, anal papillae and carcass of 4th instar larvae with RT-PCR and RT-qPCR. [file 40249_2022_990_MOESM5_ESM.docx]
