# Supplementary material for: Armigeres subalbatus is a potential vector for Zika virus but not dengue virus
Source: Infect Dis Poverty. 2022 Jun 4;11:62. doi: 10.1186/s40249-022-00990-0 (PMC9166152; doi:10.1186/s40249-022-00990-0)
Supplement: Supplementary file 2 — Additional file 2: Table S1. Genetic analysis of the mosquito species isolated in Guangdong based on the COI gene. [file 40249_2022_990_MOESM2_ESM.docx]

**Additional file 2: Table S1. Genetic analysis of the mosquito species isolated in Guangdong Province based on the COI gene.**

| Description | Scientific Name | Max Score | Total Score | Query Cover | E value | Per. ident | Accession |
| --- | --- | --- | --- | --- | --- | --- | --- |
| *Armigeres subalbatus* voucher  2019-Ar-WC-6 cytochrome c oxidase  subunit I (COX1) gene, partial cds; mitochondrial | *Armigeres  subalbatus* | 1138 | 1138 | 100% | 0 | 100 | MT541036.1 |
| *Armigeres subalbatus* isolate WENCHANG  cytochrome c oxidase subunit I (COX1) gene,  partial cds; mitochondrial | *Armigeres  subalbatus* | 1138 | 1138 | 100% | 0 | 100 | MW940867.1 |
| *Culicidae sp.* Ne39M cytochrome c oxidase  subunit I (COI) gene, partial cds; mitochondrial | *Culicidae sp.  Ne39M* | 828 | 828 | 100% | 0 | 90.92 | KP845048.1 |
| *Aedes mcintoshi* isolate MAN1  cytochrome oxidase subunit I (COI) gene,  partial cds; mitochondrial | *Aedes  mcintoshi* | 800 | 800 | 100% | 0 | 90.1 | KJ940647.1 |
| *Aedes lineatopennis* isolate 35-3  cytochrome oxidase subunit I (COI)  gene, partial cds; mitochondrial | *Aedes  lineatopennis* | 791 | 791 | 98% | 0 | 90.13 | KT358468.1 |
