# Supplementary material for: Armigeres subalbatus is a potential vector for Zika virus but not dengue virus
Source: Infect Dis Poverty. 2022 Jun 4;11:62. doi: 10.1186/s40249-022-00990-0 (PMC9166152; doi:10.1186/s40249-022-00990-0)
Supplement: Supplementary file 3 — Additional file 3: Table S2. The infections of ZIKV in Ar. subalbatus. [file 40249_2022_990_MOESM3_ESM.docx]

**Additional file 3. Table S2. The infections of ZIKV in *Armigeres subalbatus*.**

| Tissue | Infections (no. positive/ no. total (%)) | | | | |
| --- | --- | --- | --- | --- | --- |
|  | 4 dpi^#^ | 7 dpi | 10 dpi | 14 dpi | 21 dpi |
| Midgut | 13/72 (18.0) | 20/72 (27.8) | 8/72 (11.1) | 10/72 (13.9) | 16/72 (22.2) |
| Ovary | 0/72 (0) | 6/72 (8.3) | 4/72 (5.6) | 3/72 (4.2) | 7/72 (9.7) |
| Salivary gland | 0/72 (0) | 0/72 (0) | 3/72 (4.2) | 3/72 (4.2) | 4/72 (5.6) |

^#^dpi (days post inoculation).

IR (Infection rate) = the number of positive midguts/the total number of midguts (100%).

DR (Dissemination rate) = the number of positive ovaries/the number of positive midguts (100%).

TR (Transmission rate) = the number of positive salivary glands/the total number of midguts (100%).
