# Supplementary material for: Armigeres subalbatus is a potential vector for Zika virus but not dengue virus
Source: Infect Dis Poverty. 2022 Jun 4;11:62. doi: 10.1186/s40249-022-00990-0 (PMC9166152; doi:10.1186/s40249-022-00990-0)
Supplement: Supplementary file 6 — Additional file 6: Table S3. Infection rate of DENV-2 in larvae of Ar. subalbatus. [file 40249_2022_990_MOESM6_ESM.docx]

**Additional file 6: Table S3. Infection rate of DENV-2 in larvae of *Ar. subalbatus*.**

| Infection method | Viral titer  (pfu/ml) | Experiment repetition | No. larvae tested pools* | No. Positive pools |
| --- | --- | --- | --- | --- |
| Continuous  addition | 10^4^ | 1 | 13 | 0 |
|  |  | 2 | 13 | 0 |
|  |  | 3 | 15 | 0 |

* Ten larvae per pool.
